# Preventing Cardiovascular Complications in Type 1 Diabetes: The Need for a Lifetime Approach

**DOI:** 10.3389/fped.2021.696499

**Published:** 2021-06-09

**Authors:** Scott T. Chiesa, M. Loredana Marcovecchio

**Affiliations:** ^1^Institute of Cardiovascular Science, University College London, London, United Kingdom; ^2^Department of Paediatrics, University of Cambridge, Cambridge, United Kingdom

**Keywords:** type 1 diabetes, cardiovascular, complications, adolescence, risk factors

## Abstract

Cardiovascular disease (CVD) remains the main cause of morbidity and mortality in individuals with type 1 diabetes (T1D). Adolescence appears to be a critical time for the development of early subclinical manifestations of CVD, with these changes likely driven by a deterioration in glycemic control during the progression through puberty, combined with the emergence of numerous other traditional cardiometabolic risk factors (e.g., hypertension, dyslipidemia, smoking, alcohol use, obesity, etc.) which emerge at this age. Although hemoglobin A1C has long been the primary focus of screening and treatment strategies, glycemic control remains poor in youth with T1D. Furthermore, screening for cardiovascular risk factors—which are often elevated in youth with T1D—is suboptimal, and use of pharmacological interventions for hypertension and dyslipidemia remains low. As such, there is a clear need not only for better screening strategies for CVD risk factors in youth, but also early interventions to reduce these, if future CVD events have to be prevented. Accumulating evidence has recently suggested that early increases in urinary albumin excretion, even within the normal range, may identify adolescents with T1D who are at an increased risk of complications, and results from pharmacological intervention with statins and ACE inhibitors in these individuals have been encouraging. These data join a growing evidence highlighting the need for a whole-life approach to prevention starting from childhood if efforts to improve CVD outcomes and related mortality in T1D are to be maintained.

## Introduction

Type 1 diabetes (T1D) is a key public health concern, because of the growing incidence and the increased morbidity and mortality associated with this chronic condition (1, 2). Recent estimates from the International Diabetes Federation indicate that worldwide there are over 1 million individuals younger than 19 years living with T1D, with around 100,000 new cases every year in this age group (3). Concern has been raised about the increasing incidence of T1D particularly in very young children (2), as this can lead to higher rates of long-term complications such as retinal and kidney disease, neuropathy, and cardiovascular disease (CVD)—all of which have a negative impact on the prognosis of young people (4).

Although over the last decades there have been key advances in diabetes management, T1D remains a major cause of morbidity, reduced quality of life and loss of productivity (5). In addition, premature mortality in individuals with T1D still exceeds that of the background population by 2–4 fold (6–8). This burden is largely due to vascular complications, with CVD being the leading cause of mortality (9, 10). While significant improvements in the clinical management of T1D have made impressive inroads into these troubling figures in recent decades, ever-increasing population levels of obesity and its accompanying metabolic derangements mean that a failure to adequately address more “traditional” CVD risk factors may stall future progress (11).

The purpose of this review is to offer an update on cardiovascular risk in youth with T1D with a focus on (1) non-glycemic risk factors for CVD; (2) new markers/measures for the early detection of CVD, (3) new intervention strategies. Relevant research studies, primarily published in the last 10 years, and including pediatric populations, are reviewed.

## The Burden Associated With CVD in T1D

Premature atherosclerosis is the main cause of excess mortality in individuals with T1D, with a standardized mortality attributable to CVD of 5.7 for men and 11.3 for women (12). Individuals with T1D experience an earlier onset of cardiovascular events and a higher related mortality compared to their peers without diabetes, and women with T1D are at higher risk than men (13, 14).

The incidence of major coronary artery disease events in young adults (aged 28–38 years) with T1D is around 1% per year and increases over 3% per year after age 55 years (4). Evidence of premature atherosclerosis may be evident in as many as 50–70% individuals with T1D by the age of 45 years, and a significant proportion of young people will have clinical cardiovascular risk factors already detectable by the age of 12–19 years (15).

Great strides been made in recent decades to lower mortality in people with T1D (16), with most of this success attributed to improvements in glycemic control due to better insulin regimens along with the introduction of continuous subcutaneous insulin infusion and continuous glucose monitoring, and more recently hybrid closed loop systems (17). Nevertheless, individuals with T1D still experience significant excess morbidity and mortality, as evidenced by a recent Danish study assessing mortality in children and young adults (age 1–39 years) in which a 7-fold increase in all-cause mortality and 11-fold increase in cardiovascular mortality was observed compared to age-matched individuals without diabetes (18).

## Adolescence is a Critical Time for the Onset of Cardiovascular Disease

**Figure 1:**
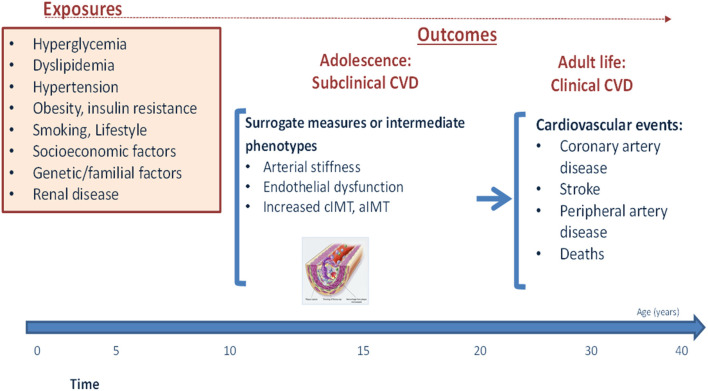
Natural history of cardiovascular disease in youth with Type 1 diabetes.

Although the clinical manifestations of CVD are almost exclusively observed in later life, their incidence represents the end-result of decades-long subclinical disease process which is driven in large part by potentially modifiable lifestyle behaviors and risk factors (19) (Figure 1). In individuals with childhood-onset T1D, adolescence appears to be a particularly critical period for this process (20). This has been starkly illustrated by findings from the Swedish National Diabetes Register, in which T1D onset before the age of 10 years was associated with a loss of life expectancy of 17.7 years in women and 14.2 years in men, as compared with 10.1 and 9.4 years respectively in those with T1D onset at age 26–30 years (7). Furthermore, individuals with childhood-onset T1D were found to have a 30-times higher CVD risk than the general population (7). Further data from the US National Health Interview Survey support this, indicating that whereas deaths related to vascular complications declined over time in adults, they remained almost unchanged in young people with T1D aged 22–44 years (21). This higher risk of CVD in people with an early onset of T1D is likely related to a longer duration of risk factor exposure in individuals diagnosed in childhood, alongside a potentially more aggressive disease pathogenesis (22).

While suboptimal glycemic control likely plays a major role in this elevated risk, it is now well-established that early and sustained exposure to “traditional” CVD risk factors such as high BMI, blood pressure and cholesterol, physical inactivity, poor diet, and smoking (amongst others) likely accelerate the atherosclerotic disease process and further contribute to future CVD risk (19, 23) (Figure 1). These adverse risk factors frequently track across the lifespan, with individuals who are exposed to a high-risk factor burden already during adolescence more likely to carry this increased burden into later-life (24). This early and cumulative exposure to multiple risk factors throughout adolescence may therefore be particularly damaging in individuals with childhood-onset T1D, especially as it occurs alongside progressively worsening HbA1c levels (25). In support of this, multiple studies in individuals with T1D have demonstrated that it is during this period of life that the first signs of vascular damage often appear (26). These early vascular complications may be particularly aggressive in those with poor glycemic control (27), and have been shown to increase even further in those additionally exposed to other traditional risk factors (28).

The most common method for assessing these early subclinical manifestations are through the use of surrogate markers such as increased arterial wall thickness (carotid intima-media thickness, cIMT), accelerated arterial stiffening (pulse wave velocity, PWV), or endothelial dysfunction (flow mediated dilation, FMD) (15, 29). A recent systematic review and meta-analysis including 23 studies on cIMT and carotid-femoral PWV showed higher cIMT and PVW in youth with T1D than matched controls (30).

With recent evidence demonstrating a remarkable ability for even modest reductions in risk factors to substantially reduce cardiovascular events when sustained across the life-course (31), it is clear that efforts to mitigate risk factor exposure should start as early as possible and continue during the transition through adulthood, if maximal lifetime benefits are to be achieved.

## Non-Glycemic CVD Risk Factors in Youth With T1D: A Burgeoning Concern

Poor glycemic control is the primary modifiable risk factor for CVD in T1D, as clearly highlighted by the results of the DCCT/EDIC studies and other more recent studies (32). However, other traditional cardiometabolic risk factors such as obesity, hypertension, and dyslipidemia; lifestyle factors such as smoking and physical inactivity, and comorbidities such as microalbuminuria, hyperfiltration/delcline in renal function can all independently and additively contribute to overall CVD risk (32).

### Cardiometabolic Risk Factors

The increased prevalence of obesity in the past three decades has particularly alarmed the global health community, with up to 60% of the world's population now projected to become overweight or obese within 10 years if current trends continue (33). Worryingly, the most rapid increases in obesity during this time have been in the young—the vast majority of whom will go onto become obese adults (34). Although individuals with T1D were traditionally considered to be a relatively lean population, recent evidence has shown that obesity rates in this group are now similar or possibly even higher than in the general population, with an alarmingly high prevalence particularly in children and adolescents (35). Indeed, recent data based on large registries from the US, Australia and Europe have confirmed that overweight and obesity are common among youth with T1D at rates of 9–20% (36). Given the known causal relationship between obesity, its accompanying sequelae of cardiometabolic derangements, and CVD; these data suggest that recent improvements in CVD morbidity/mortality, which have been largely attributed to improved glycemic control, could now be at risk of stalling or even being reversed due to the emergence of these additional emerging risk factors (11).

Unfortunately, evidence suggests that youth with T1D are disproportionately affected by these risk factors when compared to their healthy peers, and the presence of many of these factors are therefore already common among youth with T1D. For example, recent studies have suggested that up to 60% of children may show evidence of cardiometabolic risk factors around the time of diagnosis (37, 38), and around 86% of individuals may have one and 14–45% more than two cardiovascular risk factors by the time they reach adolescence (15). Similar findings have been reported from the SEARCH study, in which 7% of T1D youth had two or more CVD risk factors and 1.7% had three or more. In these individuals, 26% participants were overweight, 14% obese, 13% hypertensive, and 29% dyslipidemic (32). In longitudinal follow-up, while the number of these risk factors did not significantly change over the 10-year study period [2002–2012], a strong relationship was found between BMI and CVD risk factors, and a clustering of CVD risk factors was associated with high rates of multiple vascular complications (39). Likewise, in a recent large Italian study, assessing 2021 young people with T1D aged 2–18 years, CVD risk factors were confirmed to be common, with 32.5% showing one CVD risk factor and 6.7% two risk factors (40).

### Lifestyle Risk Factors

In addition to cardiometabolic derangements, lifestyle factors such as smoking, alcohol, sedentary behavior, and stress can also contribute to CVD (15). Among teenagers with T1D, 10% reported alcohol consumption, 10% were smokers and 6% reported both alcohol and cigarette use (41). Both smoking and alcohol in youth are associated with suboptimal glycemic control, alongside a higher prevalence of other CVD risk factors (41).

### Emerging Risk Factors

DKD is an independent risk factor for cardiovascular morbidity and mortality (42, 43). Albuminuria, the hallmark of DKD, is associated with two to four times greater risk for CVD and death (44), and there is strong evidence that urinary albumin excretion is a continuous CVD risk factor (45). Longitudinal studies of adolescents with T1D recruited to the Oxford Regional Prospective Study, Nephropathy Family Study and the Adolescent Type 1 Diabetes cardio-renal Intervention Trial (AdDIT), have highlighted these observations (26). In these young cohorts, increases in the urinary ACR may occur as early as 1 year from T1D diagnosis in those who progress to microalbuminuria or macroalbuminuria (46). Furthermore, these modest increases in albumin excretion are not only linked to early risk for the development of microalbuminuria (46) but also to CVD risk factors such as carotid intima-media thickness, flow-mediated dilation, C-reactive protein, and over time changes in blood pressure (47, 48). Thus, urinary ACR is an important CVD risk marker in addition to traditional predictive risk factors in T1D. In addition, decline in glomerular filtration rates, which can occur already in youth with T1D, is an additional risk factor for CVD (49).

## How Best to Tackle CVD Risk Factors During Adolescence?

Collectively, the above findings have led to guidelines from organizations such as the International Society for Pediatric and Adolescent Diabetes and American Diabetes Association to recommend screening for CVD risk factors (primarily dyslipidemia, high blood pressure, obesity, and smoking) starting from the age of 10–11 years (50). Despite these recommendations, however, strategies on how to appropriately address these once identified remain suboptimal.

During adolescence, the primary focus of management of vascular complications risk is to improve glycemic control by intensifying insulin therapy (50). However, despite advances in insulin treatment, over 75% adolescents do not reach recommended targets for HbA1c (25).

In addition to issues with glycemic control, many adolescents also do not meet targets for blood pressure, cholesterol or BMI (15), potentially compounding the excess risk experienced in T1D that arises from high blood glucose levels. Guidelines recommend lifestyle interventions as first steps to control these risk factors before implementing drug interventions (50), despite a lack of strong evidence for their effectiveness. Weight management in youth with T1D presents its own challenges, with fear of hypoglycemia, difficult management of glycemic control during exercise and inadequate knowledge around exercise management representing potential barriers to increased levels of physical activity (51). Furthermore, while adherence to dietary guidelines is associated with improved glycemia, youth struggle to meet strict dietary recommendations—particularly during the critical transition through adolescence when newfound freedoms from parental oversight and other social pressures often have negative effects on diet or other health behaviors (52).

Despite these difficulties, however, robust evidence exists to suggest that the adoption of several relatively simple lifestyle changes in the early years of life are likely to significantly reduce risk of CVD in later years. In the general population, evidence from the CARDIA study has shown the remarkable effect that good health behaviors in adolescence/young adulthood have on cardiovascular risk in later life, with adolescents with 5 or more healthy lifestyle behaviors (as defined by the American Heart Association and comprising blood pressure, total cholesterol, glucose/HbA1c, BMI, physical activity, diet, smoking) over 20 times more likely to have a favorable cardiovascular health profile in 20 year-follow-up compared to those with 0 healthy behaviors (53). These findings have recently been replicated in individuals with T1D for the first time, suggesting that these metrics represent straightforward goals for health promotion that may reduce CVD risk in the T1D population (54). Together, these findings highlight the need for more focus on education and training on exercise and lifestyle change in diabetes to enable the implementation of early intervention programmes to tackle poor cardiovascular health from a young age.

## Drug Interventions: Where are We?

Similar to lifestyle changes, recent studies have highlighted a ‘therapeutic inertia' related to treatment of CVD risk factors such as dyslipidemia and hypertension in youth with T1D, with low rates of use of antihypertensive or lipid-lowering medications even where there is an indication for their use (55, 56). Data from T1D registries from the United States and Germany/Austria have confirmed that most young patients are inadequately treated for hypertension and dyslipidaemia (57). Few young adults aged <26 years receive antihypertensive (3–5%) and lipid-lowering (1–3%) medications, highlighting the need for improved diabetes and cardiovascular risk management strategies in T1D.

Survey data suggest that clinicians endorse lifestyle recommendations for initial management of dyslipidemia and hypertension in 83–99% of cases, although only 6–17% of them believe that these are effective. In contrast, medications are rarely prescribed (58), partly due to limited data and guideline recommendations for the use of common drugs such as statins and ACE inhibitors use in adolescents with T1D.

### Statins and ACE-Inhibitors in Adolescents With T1D

AdDIT was the first large randomized clinical trial evaluating the use of ACE inhibitors and statins during adolescence to protect against T1D vascular complications (59). The trial showed that statins can reduce exposure to high lipid levels and ACE-inhibitors can reduce new cases of microalbuminuria; changes that could potentially lead to protection against future complications (59). In addition, a recent analysis of a subgroup of the trial population showed that ACE inhibitors improved endothelial function (assessed by flow mediated dilation) in high-risk adolescents transitioning through puberty, and may therefore offer long-term cardio-renal benefit during this potentially critical time period for the development of CVD (48).

The trial also provided reassuring data on the safety profile of both drugs in this age group (59). Overall adherence during the 2–4 year trial period was 75–80%, although it deteriorated over time, therefore highlighting the need of strategies to reinforce adherence to gain the maximum benefit from these interventions (60).

Participants from the AdDIT study are now entering their 2^nd^ to 3^rd^ decade from T1D diagnosis, a time when more pronounced arterial changes begin to emerge. Current follow-up of the study cohort up to 5 years from the end of the original trial, including detailed cardiovascular phenotyping, will provide invaluable information on the potential impact of cumulative exposure to acquired modifiable risk factors on CVD progression. The study will also determine whether a reduction in these risk factors during adolescence because of statin/ACE-inhibitors therapy results in long-term benefits for CVD health.

### Metformin in Youth With T1D

Metformin is another potential treatment in youth with T1D. Although there is no strong evidence in terms of glycemic control following metformin treatment, its use has been shown to lead to small reductions in total daily insulin dose and BMI in youth with T1D (61). Furthermore, recent studies have shown a beneficial effect on insulin sensitivity and CVD risk profile. In one study, a 3-month intervention with metformin led to greater improvements in insulin sensitivity compared to placebo (62), while a second smaller study on 16 youth with T1D showed that 12-month treatment with metformin improved vascular smooth muscle function (63). In a more recent mechanistic study, 48 youth with T1D (aged 12–21 years) underwent a hyperinsulinemic euglycemic clamp and assessment of MRI-derived measures of aortic and carotid vascular health, before and after 3 months of metformin or placebo therapy. Treatment with metformin was associated with improved insulin sensitivity and aortic and carotid wall measures (64). Additional larger studies are required to confirm these findings and provide additional evidence before metformin could be recommended as an adjunct therapy in T1D.

### Other Adjunct Medications in Youth With T1D

Non-insulin therapies, such as sodium-glucose co-transporter 2 (SGLT2) inhibitors, glucagon-like peptide-1 receptor agonist and dipeptidyl peptidase-4 inhibitors, have been investigated to try to improve glycemic control, as well as for their potential effect in CVD risk factors (65).

A large body of evidence support a positive effect of SGLT2-inhibitors on cardiovascular and renal outcomes in adults with diabetes, although data for the pediatric population are lacking (65). While these medications appear promising, the risk for euglycemic diabetic ketoacidosis is still a potential limiting factor to consider before a wide implementation in the pediatric population (65).

## Motivational and Psychological Interventions

Data based on semi-structured interviews have clearly shown poor awareness of complications among adolescents as well their reluctance to know about them (66). Even when patients are aware, however, a combination of insufficient support for how to implement lifestyle changes, lack of confidence to follow these changes, and lack of knowledge on other means for controlling blood pressure and lipid levels may all limit uptake of preventative measures (67). More effort is therefore required to not only raise awareness about complications in youth, but also to develop better ways to quantify and communicate this risk to patients and their families. To achieve this, potential barriers limiting adherence to weight management and lifestyle strategies should be identified and addressed (51), and reassurance about medical treatment of dyslipidemia and hypertension should be provided to both young patients and their parents.

Properly addressing physical activity, nutrition, pharmacotherapy, and psychosocial factors while emphasizing weight management may improve CVD risk factors and avoid their persistence during adulthood.

## Unanswered Questions and Future Directions

### Early Prediction

There is still a need for improved ways to identify people at risk at an early stage when pathology may be amenable to interventions.

Early screening for abnormal ACR may provide a valuable tool to identify adolescents at high risk for CVD in clinical practice. Risk stratification using urinary albumin excretion along with other traditional and new risk factors during early adolescence may be critical for the early identification of patients at risk and to guide the implementation of preventive and treatment strategies.

### Early Prevention/Better Interventions

There is an urgent need to identify new targets for interventions to prevent CVD complications, and this can only be achieved through a better understanding of the mechanisms and key players implicated in the development and progression of vascular complications.

While the AdDIT trial has provided evidence that early interventions with ACE-Inhibitors and statins are both safe and effective in reducing exposure to CVD risk factors during adolescence, the impact of these early interventions on long-term cardiovascular outcomes needs to be further explored. For any intervention to provide maximum efficacy, strategies to promote adherence to both existing and new interventions will be required—especially given known issues surrounding adherence levels common during the transition from adolescence to adulthood. In addition, lifestyle interventions need to be more widely promoted and made easy to be implemented for youth with T1D.

## Conclusions

Despite significant improvements in the management of T1D during the past decades, vascular complications remain a major concern. Efforts to improve vascular outcomes and mortality in T1D should be a whole-life approach starting from childhood. Early investment in the understanding and care of youth with T1D from childhood will have substantial long-term benefits in terms of complications, quality of life, and life expectancy.

## Author Contributions

SC and MM reviewed literature and worked together on the manuscript draft and approved final version. Both authors contributed to the article and approved the submitted version.

## Conflict of Interest

The authors declare that the research was conducted in the absence of any commercial or financial relationships that could be construed as a potential conflict of interest.
